# Multiplexity of human brain oscillations as a personal brain signature

**DOI:** 10.1002/hbm.26466

**Published:** 2023-09-05

**Authors:** Stavros I. Dimitriadis, B. Routley, David E. J. Linden, Krish D. Singh

**Affiliations:** ^1^ Cardiff University Brain Research Imaging Centre, School of Psychology Cardiff University Cardiff Wales UK; ^2^ MRC Centre for Neuropsychiatric Genetics and Genomics, Division of Psychological Medicine and Clinical Neurosciences, School of Medicine Cardiff University Cardiff Wales UK; ^3^ Department of Clinical Psychology and Psychobiology University of Barcelona Barcelona Spain; ^4^ School for Mental Health and Neuroscience, Faculty of Health Medicine and Life Sciences Maastricht University Maastricht The Netherlands

**Keywords:** chronnectomics, dominant coupling modes, Multiplexity, individual fingerprint, MEG, resting‐state, signal processing, time‐varying network analysis

## Abstract

Human individuality is likely underpinned by the constitution of functional brain networks that ensure consistency of each person's cognitive and behavioral profile. These functional networks should, in principle, be detectable by noninvasive neurophysiology. We use a method that enables the detection of dominant frequencies of the interaction between every pair of brain areas at every temporal segment of the recording period, the dominant coupling modes (DoCM). We apply this method to brain oscillations, measured with magnetoencephalography (MEG) at rest in two independent datasets, and show that the spatiotemporal evolution of DoCMs constitutes an individualized brain fingerprint. Based on this successful fingerprinting we suggest that DoCMs are important targets for the investigation of neural correlates of individual psychological parameters and can provide mechanistic insight into the underlying neurophysiological processes, as well as their disturbance in brain diseases.

## Graphical abstract

We address a fundamental question of human neuroscience, whether we can discover the brain correlates of individuality. We demonstrate that people can be identified as reliable based on the multiplexity of neural activity measured with MEG at rest between brain areas located over the default‐mode, cingulo‐opercular and frontoparietal networks.

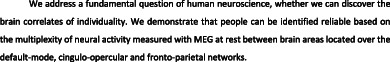

## 1 INTRODUCTION

Despite over 30 years of functional neuroimaging in humans, the correlates of individuality in the brain have not been elucidated up to now. A likely reason is that human neuroscience studies generally aggregate data across a group of subjects to reveal commonalities in brain activity and connectivity patterns or to obtain salient differences between patient and control groups, whereas the heterogeneity within each group is typically ignored.

However, even among neurologically healthy people, both brain structure (Amunts et al., 2000; Bürgel et al., 2006; Mangin et al., 2004) and function (Grabner et al., 2007; Newman et al., 2003; Rypma & D'Esposito, 1999) are highly variable. Functionally, inter‐subject variability was found to be high both for functional activation during cognitive tasks (Grabner et al., 2007; Newman et al., 2003; Rypma & D'Esposito, 1999) and for the intrinsic functional organization of the brain at rest (Mueller et al., 2013). For example, monozygotic twins only share a small variance in their local structural connectome (van Essen et al., 2013), allowing for a considerable amount of variability even between genetically identical individuals. Conversely, within individuals, structural and functional connectivity measures seem to be very stable over time (Barch et al., 2013; Yeh et al., 2016), even across several months (Finn et al., 2015; Powell et al., 2018). Brain connectomics is, therefore, an attractive tool to investigate the brain signatures of individual differences (Dimitriadis & Salis, 2017).

The motivation behind the idea of “brain fingerprinting” with neuroimaging signals is not mainly to identify the identity of individuals. The general motivation of brain fingerprinting is to capture individually specific features of the brain, and ultimately to achieve a mechanistic interpretation that can contribute to a new understanding of the biological basis of personality, characteristic biological features of individuals, and other individual traits using neuroimaging (Liu et al., 2019). The identification of the “brain‐fingerprinting” subnetwork with the use of a neuroimaging modality will moreover enhance our understanding of brain networks that are likely to be particularly vulnerable to perturbations leading to cognitive, behavioral, and psychopathological abnormalities (Finn & Todd Constable, 2016; Kanai & Rees, 2011; van den Heuvel & Sporns, 2013).

Brain fingerprinting has been studied with electroencephalography (EEG; Dimitriadis & Salis, 2017), functional magnetic resonance imaging (fMRI; Finn et al., 2015; Liu et al., 2018), diffusion MRI (dMRI; Yeh et al., 2016) and magnetoencephalography (MEG; da Silva Castanheira et al., 2021; Sareen et al., 2021). MEG arguably has the highest sensitivity to spatiotemporal fluctuations in fine‐grained activity among the noninvasive neuroimaging techniques. Compared to fMRI (Abrol et al., 2017), MEG is a more direct measure of functional connectivity that can uncover the dominant coupling modes of brain networks and the multi‐scale frequency‐dependent interactions across space and time (Engel et al., 2013).

Brain rhythms that can be detected with MEG range from the infraslow (<0.01 Hz) to ultrafast frequencies (200–600 Hz) and include at least 10 interactive oscillation classes each one with a specific frequency width ranging from the slow 4 (<0.01 Hz) up to ultra‐fast (200–600 Hz; Buzsáki et al., 2013). These brain rhythms often interact in the same brain state either within the same or across different structures, in a multiplex way that supports within‐frequencies and between‐frequencies coupling modes (cross‐frequency coupling; Engel et al., 2013; Khazipov et al., 2004). We thus propose that, for a comprehensive evaluation of network integration and its individual specificity, both phase‐to‐phase (within frequency) and phase‐to‐amplitude cross‐frequency coupling mechanisms need to be analyzed (Engel et al., 2013; Siebenhühner et al., 2016), which is the approach taken in the present study.

Our goal was to investigate if the *multiplexity* of brain communication at MEG resting‐state and in healthy individuals explored under our dominant coupling model (DoCM) model (Dimitriadis, 2018, 2021) can be served as *a personalized brain signature*. For that purpose, we trained our proposed analytic pipeline in a test–retest study and we validated the outcome of this repeat cohort in a larger MEG cohort (*N* = 183) with an unknown number of common subjects between the two for the experimenter (SID).

## 2 MATERIALS AND METHODS

### 2.1 Subjects

#### 2.1.1 Repeat scan cohort (Experiment 1)

Forty healthy subjects (age 22.85 ± 3.74 years, 15 women and 25 men) underwent two resting‐state MEG sessions (eyes open) over 2 consecutive weeks. The duration of the resting‐state condition was 5 mins. For each participant, scans were scheduled on the same day of the week and at the same time of the day. This is a test–retest study performed in CUBRIC Neuroimaging Centre with the main aim to evaluate the repeatability of various measurements that can be extracted from MEG resting‐state recordings. The study was approved by the Ethics Committee of the School of Psychology at Cardiff University, and participants provided informed consent.

#### 2.1.2 Validation cohort (Experiment 2)

The second validation cohort consists of MEG resting‐state recordings from *N* = 183 subjects (64 males and 119 females: 119 with mean age of 24.79 and SD 5.68). This large cohort is a collection of multimodal neuroimaging datasets performed in CUBRIC Neuroimaging Centre from a healthy population. The multimodal neuroimaging protocol of the repeat scan cohort is the same as the validation cohort. The duration of the resting‐state condition was 5 mins. The study was approved by the Ethics Committee of the School of Psychology at Cardiff University, and participants provided informed consent. Twenty‐two subjects of the first cohort were also in this second cohort. This partial overlap provided an additional challenge to the classification procedure.

These data were provided to SID by the study PIs (DEL and KS) without prior information as to whether there were any common subjects between the MEG repeat scan study and the normative database.

The recruitment of participants in both cohorts was inclusive to all persons without limitations by (1) sex or gender, (2) race or ethnicity, or (3) age other than as scientifically justified and as specified in enrollment inclusion and exclusion criteria.

### 2.2 MEG‐MRI recordings

Whole‐head MEG recordings were made using a 275‐channel CTF radial gradiometer system. An additional 29 reference channels were recorded for noise cancellation purposes and the primary sensors were analyzed as synthetic third‐order gradiometers. Two or three of the 275 channels were turned off due to excessive sensor noise (depending on time of acquisition). Subjects were seated upright in the magnetically shielded room. To achieve MRI/MEG co‐registration, fiduciary markers were placed at fixed distances from three anatomical landmarks identifiable in the subject's anatomical MRI, and their locations were verified afterward using high‐resolution digital photographs. Head localization was performed before and after each recording, and a trigger was sent to the acquisition computer at relevant stimulus events.

All datasets were either acquired at or down‐sampled to 600 Hz, and filtered with a 1‐Hz high‐pass and a 200‐Hz low‐pass filter. The data were first whitened and reduced in dimensionality using principal component analysis with a threshold set to 95% of the total variance. The statistical values of kurtosis, Rényi entropy, and skewness of each independent component were used to eliminate ocular, muscle, and cardiac artifacts. We estimated those metrics in a dynamic fashion adopting a sliding window mode of width 2 s with no overlapping leading to a number of 30 (per min) × 5 (mins) = 150 temporal segments. Specifically, a component was deemed artifactual if more than 20% of the total number of temporal segments (more than 30) showed all the metric values after normalization to zero‐mean and unit‐variance outside the range of [−2, +2]. The artifact‐free multichannel MEG resting‐state recordings were then entered into the beamforming analysis (see next section).

Subjects further underwent an MRI session in which a T1‐weighted 1‐mm anatomical scan was acquired, using an inversion recovery spoiled gradient echo acquisition. Both MRI and MEG recordings in both cohorts have been collected on the same day following a common multimodal neuroimaging protocol in CUBRIC Neuroimaging Centre.

### 2.3 Beamforming

An atlas‐based beamformer approach was adopted to project MEG data from the sensor level to source space independently for each brain rhythm. The frequency bands studied were δ (1–4 Hz), θ (4–8 Hz), α_1_ (8–10 Hz), α_2_ (10–13 Hz), β_1_ (13–20 Hz), β_2_ (20–30 Hz), γ_1_ (30–45 Hz), and γ_2_ (55–90 Hz). First, the coregistered MRI was spatially normalized to a template MRI using SPM8 (Weiskopf et al., 2011). The AAL atlas was used to anatomically label the voxels, for each participant and session, in this template space. The 90 cortical regions of interest (ROIs) were used for further analysis, as is common in recent studies (Hillebrand et al., 2016). Next, neuronal activity in the atlas‐labeled voxels was reconstructed using the LCMV source localization algorithm as implemented in Fieldtrip (Oostenveld et al., 2011). The MEG lead field was based on a VC model created using the boundary element method (BEM).

The beamformer sequentially reconstructs the activity for each voxel in a predefined grid covering the entire brain (spacing 6 mm) by weighting the contribution of each MEG sensor to a voxel's time series—a procedure that creates the spatial filters that can then project sensor activity to the cortical activity. Each ROI in the atlas contains many voxels, and the numbers of voxels per ROI differ. To obtain a single representative time series for every ROI, we defined a functional‐centroid ROI representative by functionally interpolating activity from the voxel time series, within each ROI, in a weighted fashion. Specifically, we estimated a functional connectivity map between every pair of source time series within each of the AALs ROIs (Equation 1) using the absolute value of the Pearson's correlation coefficient (Equation 2). We then estimated the connectivity strength of each voxel within the ROI by summing its connectivity values to other voxels within the same ROI (Equation 3) and finally, we normalized each strength by the sum of strengths (Equation 4) to estimate a set of weights within the ROI that sum to a value of 1. Finally, we multiplied each voxel time series with their respective weights and we summed across them to get a representative time series for each ROI (Equation 5). The whole procedure was applied independently to every quasi‐stable temporal segment derived by the settings of temporal window and stepping criterion.

The following Equations (1), (2), (3), (4), (5) demonstrated the steps for this functional interpolation.

(1) $${\mathrm{ROI}}^{\mathrm{map}}\in R^{\text{voxels}\times \text{samples}},\text{voxels}\in \mathrm{no}\;\text{of voxel timeseries within each}\;\mathrm{ROI}$$

(2) $$S^{\text{Voxels}}=\sum_{k=1}^{\text{Voxels}}\sum_{l=k+1}^{\text{Voxels}}\mathrm{abs}(\text{corr}\left({\mathrm{ROI}}_{k}^{\mathrm{map}}\left(t\right),\text{corr}\left({\mathrm{ROI}}_{l}^{\mathrm{map}}\left(t\right)\right)\right),S^{\text{Voxels}}\in \mathrm{ROI}\;X\;\mathrm{ROI}$$

(3) $$\;{\mathrm{SS}}_{k}=\sum_{k=1}^{\text{Voxels}}\text{corr}\left(k,:\right),\;\mathrm{SS}\in 1\times \mathrm{ROI}$$

(4) $$W_{k}=\frac{{\mathrm{SS}}_{k}}{\sum_{k=1}^{\text{Voxels}}{\mathrm{SS}}_{k}}$$

(5) $${\mathrm{ROI}}^{\text{activity}}=\sum_{k=1}^{\text{Voxels}}{\mathrm{ROI}}_{k}^{\text{time series}}*W_{k}$$

#### 2.3.1 MEG dynamic source connectivity analysis

A dynamic connectivity analysis, based on a sliding‐window approach, was applied to eight conventionally defined frequency bands: δ (0.5–4 Hz); θ (4–8 Hz); α_1_ (8–10 Hz); α_2_ (10–13 Hz); β_1_ (13–20 Hz), β_2_ (20–30 Hz), γ_1_ (30–45 Hz) and γ_2_ (55–90 Hz). Band‐limited brain activity was derived by applying a third‐order Βutterworth filter (in zero‐phase mode). We quantified the brain source network, employing two types of interactions and adopting properly defined connectivity estimators: (a) intra‐frequency phase coupling within each of the eight frequencies was estimated using the imaginary part of the phase locking value (iPLV; Dimitriadis et al., 2017; Dimitriadis & Salis, 2017); (b) cross‐frequency coupling (CFC), namely phase‐to‐amplitude coupling (PAC) between 28 possible pairs of frequencies was defined with the PAC estimator (Dimitriadis & Salis, 2017). The strength of the connections estimated with the two adopted connectivity estimators (iPLV/PAC) ranged from 0 to 1. The derived quantities are tabulated in a 90 × 90 matrix, called hereafter the “functional connectivity graph” (FCG), in which each element conveys the strength of iPLV/PAC for each pair of cortical sources. The aforementioned procedure produced 8 + 28 = 36 FCGs for each participant, in each sliding window. To further clarify the total amount of coupling modes, we estimated eight within‐frequency coupling modes (one per frequency), and 8 × 7/2 = 28 cross‐frequency coupling modes per pair of ROIs and per sliding mode. This procedure produces 36 coupling modes per pair of ROIs and per sliding mode.

**FIGURE 1:**
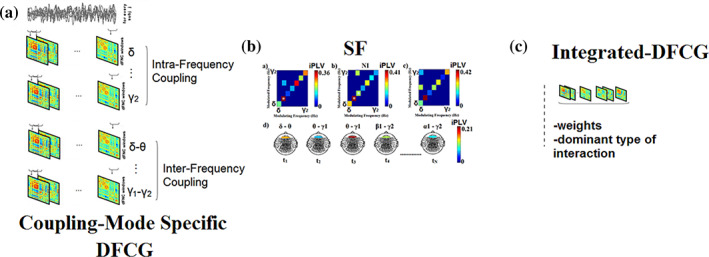
Construction of integrated dynamic functional connectivity graphs (iDFCG). (a) We constructed one DFCG per coupling mode for both within‐frequency coupling and cross‐frequency coupling modes (36 in total). Similarly, we constructed 10,000 surrogates DFCG per coupling mode, and assigned a *p*‐value per each of the 36 coupling modes for every pair of ROIs within a temporal segment. An example of the first three temporal segments from the first subject of the first cohort is illustrated in (b). From this process, we can untangle if two brain regions are functionally connected and if so, which is the preferred dominant coupling mode. (c) The outcome of the surrogate analysis is an iDFCG that preserves both the weight and the dominant type of interaction (SF ‐ Statistical Filtering).

We adopted a sliding‐window of 1 s moving every 100 ms to capture, in more detail, possible transitions of dominant intrinsic coupling modes between consecutive windows (see Sections 5 and 6 for the optimization strategy of the width of the temporal window and the stepping criterion). The whole approach led to 2991 [(300 s – 1)/0.1 + 1] time‐varying FCGs for each participant and session. For each participant and for each connectivity estimator, 4D dynamic functional connectivity graphs were derived, each with dimension: (modes:8 + 28) × 2991 (temporal segments) × 90 (ROIs) × 90 (ROIs). Table 1 summarizes the derived dynamic graphs and their dimension for each subject (see also Figure 1). Our methodology has already been demonstrated and validated in several functional neuroimaging studies (Dimitriadis, 2018, 2021; Dimitriadis & Salis, 2017).

**TABLE 1:** Dimensions and information tabulated in the dynamic functional connectivity graphs.

	Dimensions	Directed	Within frequencies	Between frequencies
iPLV	8 × 2991 × 90 × 90		✓	
PAC	28 × 2991 × 90 × 90	✓		✓

#### 2.3.2 Surrogate MEG source connectivity analysis

To estimate the statistical significance of the iPLV/PAC‐interactions, which were estimated within frequencies and for every pair of frequencies, between all possible pairs of 90 virtual sensors, and at each successive sliding window, we employed surrogate data (Dimitriadis, 2021). Surrogate data analyses determined: (a) if a given iPLV/PAC value differed from what would be expected by chance alone, and (b) if a given nonzero iPLV/PAC indicated coupling that was, at least statistically, nonspurious.

Significant iPLV values were determined after calculating iPLV for *r*_s_ = 10,000 surrogates for each connection derived by selecting a random time‐point from the amplitude time series of one of the two virtual sources and then exchanging the order of the two segments that were created (Dimitriadis, 2018, 2021; Dimitriadis & Salis, 2017). Similarly, significant PAC values were determined after calculating PAC for *r*_s_ = 10,000 surrogates for each connection derived by selecting a random time‐point from the amplitude time series (high‐frequency) and then exchanging the two ordered segments.

For every time window, virtual sensor‐pair, and pair of frequencies, we tested the null hypothesis H_0_ that the observed PAC value came from the same distribution as the distribution of surrogate PAC‐values. Ten thousand surrogate time‐series *As* ^*HF*(*t*)^ were generated by cutting at a single point at a random location the amplitude A time series (high‐frequency, HF) and exchanging the two resulting time courses (Aru et al., 2015). Repeating this procedure produced a set of surrogates with minimal distortion of the original amplitude dynamics and impact on the nonstationarity of brain activity as compared to either merely shuffling the time series or cutting and rebuilding the time series in more than one time point. With this aforementioned approach, the nonstationarity of the brain activity as captured from the source time series is less affected compared to circularly permuted phase time series (low‐frequency) for PAC relative to amplitude series (high‐frequency for PAC) and the phase of the time series for iPLV. This procedure ensures that the observed and surrogate indices share the same statistical properties. The amplitude distribution and Fourier spectra of original time series and surrogate time series are identical, the autocorrelation functions, the means, and standard deviations of amplitude distributions are also identical.

For each subject and condition, the surrogate PAC (^s^PAC) was computed. We then determined a one‐sided *p*‐value expressing the likelihood that the observed PAC value could belong to the surrogate distribution and corresponded to the proportion of “surrogate”’ PAC^s^ which was higher than the observed PAC value (Dimitriadis, 2018, 2021; Dimitriadis & Salis, 2017). PAC values associated with statistically significant *p* values were considered unlikely to reflect signals not entailing PAC coupling.

Similarly, for each subject and condition, the surrogate iPLV (^s^iPLV) was computed. We determined a one‐sided *p*‐value expressing the likelihood that the observed iPLV value could belong to the surrogate distribution and corresponded to the proportion of “surrogate”’ iPLV^s^ which was higher than the observed iPLV value (Dimitriadis, 2018, 2021; Dimitriadis & Salis, 2017). iPLV values associated with statistically significant *p* values were considered unlikely to reflect signals not entailing iPLV coupling.

After obtaining a *p*‐value per pair of MEG sources at every temporal segment and for each of 36 intra and inter‐frequency coupling modes, we corrected for multiple comparisons (*p* < 0.001; Bonferroni correction, *p*′ < *p*/36). The false discovery rate (FDR) method (Benjamini & Hochberg, 1995; Dimitriadis, 2018, 2021; Dimitriadis & Salis, 2017) was employed to control for multiple comparisons across the whole network based on the identified DoCM with the expected proportion of false positives set to *q* ≤ 0.01. Finally, the PAC mode that characterized a specific pair of frequencies was determined based on the highest, statistically significant PAC value from surrogates. Then, we compared the Bonferroni corrected *p* values for both {*i*,*j*} and {*j*,*i*} pairs of brain areas and we assigned to every pair of ROIs the type and strength of functional coupling corresponding to the lowest *p*‐value or in the case of equal *p*‐value, the one with the highest functional strength. We analyzed the resulting dynamic functional connectivity graphs as undirected.

The aforementioned statistical test is important to detect the dominant intrinsic coupling mode between every pair of virtual sources across each temporal segment. In that case, our method assigned to every pair of ROIs, the preferred type of interaction which can be from any of the 36 different coupling modes (8 intra‐frequency and 28 cross‐frequency pairs; see Figure 1b).

**FIGURE 2:**
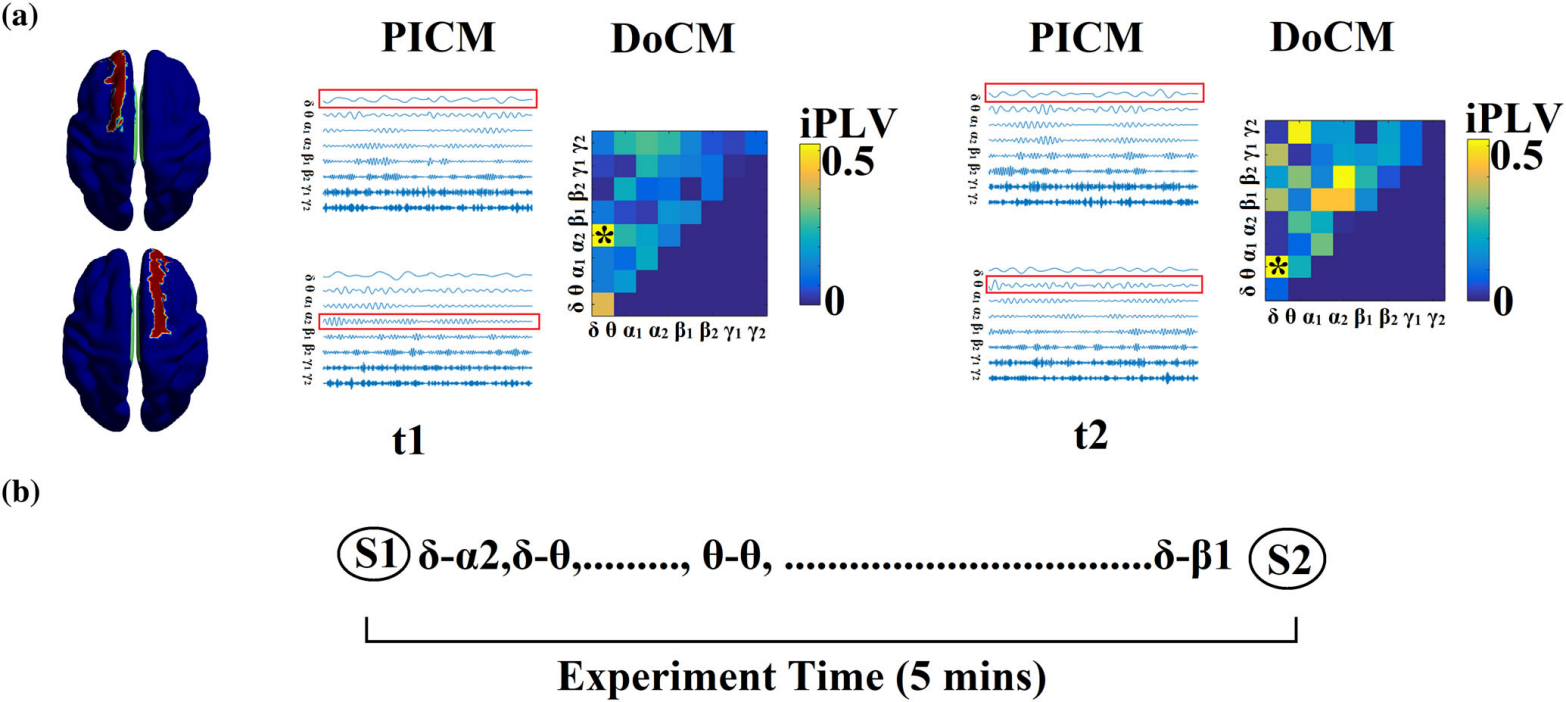
Determining Dominant Intrinsic Coupling Modes (DoCM). (a) Schematic illustration of the approach employed to identify the DoCM between two AAL atlas ROIs (left superior frontal gyrus, right superior frontal gyrus) for two consecutive 1 s sliding time windows (*t*_1_, *t*_2_) during the resting‐state MEG recording. In this example, the functional interdependence between band‐passed signals from the two virtual sensors was indexed by imaginary Phase Locking (iPLV). In this manner, iPLV was computed between the two virtual sensors either for same‐frequency oscillations (e.g., δ to δ) or between different frequencies (e.g., δ to θ; Potential Intrinsic Coupling Modes [PICM]). Statistical filtering, using surrogate data for reference, was employed to assess whether each iPLV value was significantly different from chance. During *t*_1_ the DoCM reflected significant phase locking between δ and α_2_ oscillations (indicated by red rectangles) whereas during *t*_2_ the dominant interaction was found between δ and θ oscillations. (b) Burst of DoCM between left superior frontal gyrus and right superior frontal gyrus. This packeting can be thought to group the “letters” contained in the DoCM series to form a neural “word.”, representing a possible integration of many DoCMs (Leinekugel et al, 2002).

The proposed integration model assumes that if two brain areas are communicating then this should be realized via a preferred dominant coupling mode. In Figure 2, we illustrated an example of how this model worked for a pair of virtual sources in two consecutive temporal segments. From 36 potential coupling modes, finally, we concluded to a dominant coupling mode (either intra or cross‐frequency coupling) or none.

The detection of the dominant coupling mode per pair of MEG sources is given in Figure 2b. Practically, the statistical surrogate analysis can lead to three conditions: (a) only one frequency or frequency pair met the statistical thresholding criterion, (b) in the case of two frequencies or frequency pairs both exceeding the statistical threshold, the one with the highest iPLV/PAC value was identified as the characteristic iPLV/PAC mode for this pair of virtual sensors at that particular time window and (c) if none of the within frequency or cross‐frequency pairs exceed the statistical threshold, a value of zero was assigned to this pair of virtual sensors so there is no identified characteristic coupling mode. The selection of the maximum iPLV/PAC value in the (b) condition can be adopted as a solution in the case of more than one surviving frequency and/or frequency pair since both iPLV/PAC are quantified based on the same formula. Finally, for each participant, the resulting ^TV^iPLV/PAC profiles constituted a 3D array of size [2991 (temporal segments) × 90 (sources) × 90 (sources)] that tabulated the functional coupling strength. The identity of prominent frequencies or frequency pairs for every pair of sources) at each time window was finally stored in a second 3D array of size [2991 × 90 × 90]. In the latter array, significant iPLV/PAC interactions were indicated by an integer number ranging from 1 up to 36 (1 for δ‐δ coupling, 2 for θ‐θ coupling,…, 8 for γ_2_‐ γ_2_,…,36 for β_2_‐γ_2_), with zeros indicating nonsignificant iPLV/PAC interactions. The procedure of statistical filtering is demonstrated in Figure 1c. DoCM has been already established in our previous studies (Dimitriadis, 2018, 2021; Dimitriadis & Salis, 2017).

#### 2.3.3 Dynamic reconfiguration of dominant coupling modes

**FIGURE 3:**
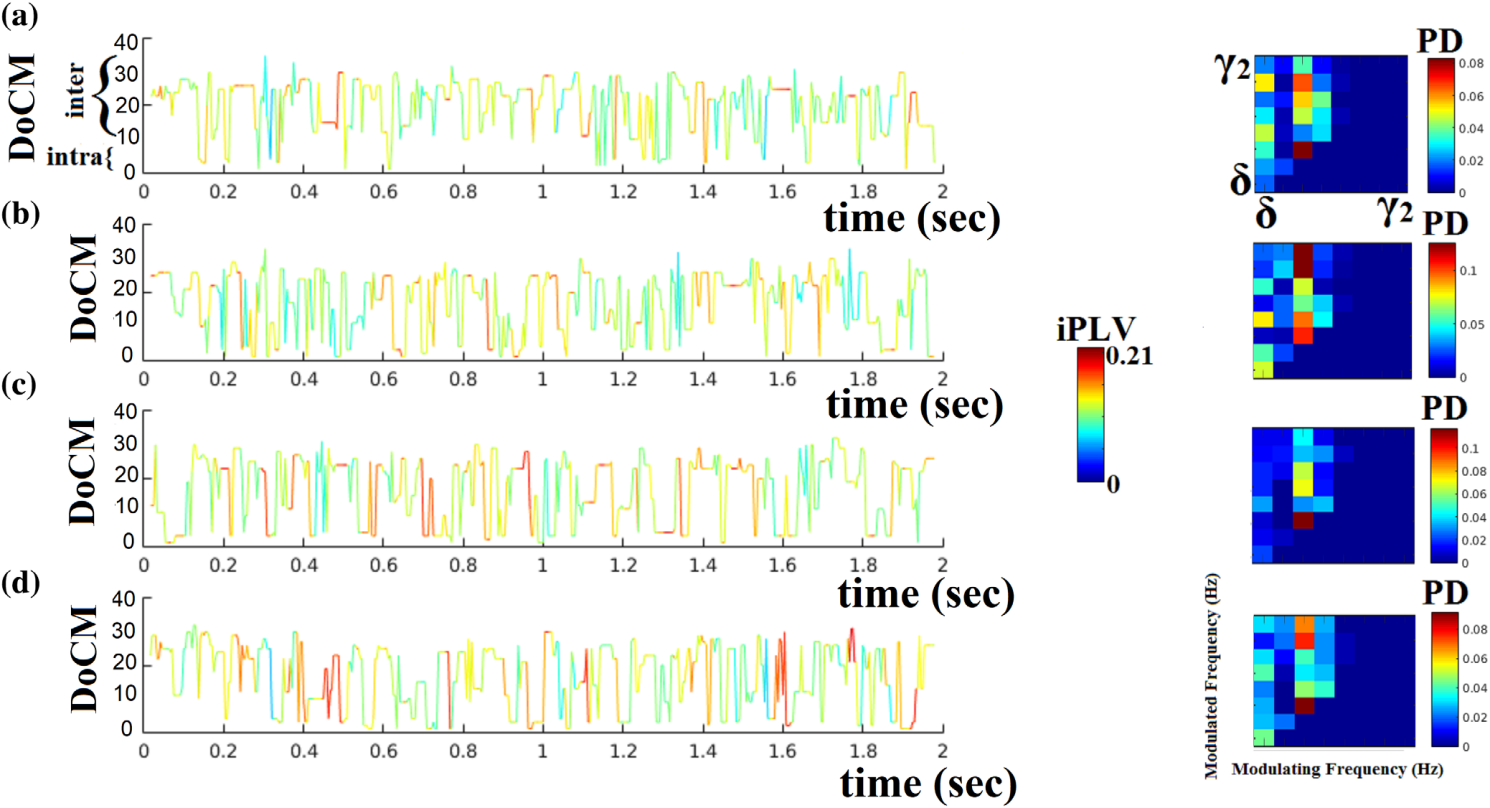
Dynamic reconfiguration of dominant coupling modes for four pairs of ROIs. (a) Right frontal‐superior‐orbital–right parietal‐superior, (b) left frontal‐middle–right parietal‐inferior, (c) left frontal‐middle–right frontal‐middle, and (d) left temporal superior–left frontal superior for subject 1. (a) In the left subplot, color represents the strength of iPLV coupling while the height of the fluctuated time series (*y*‐axis) codes the dominant intrinsic coupling mode (DoCM) over 36 possible options (8 for intra‐frequency and 28 for cross‐frequency coupling). (b) The 2D matrix is a comodulogram that tabulates the probability distribution (PD) of each dominant coupling mode across the time series presented in (a). For each time series, we plotted the comodulograms which tabulate the probability distribution of dominant coupling modes across intra (main diagonal) and cross‐frequency coupling (off‐diagonal). The total sum of the probability distribution is equal to 1. The horizontal axis refers to the modulating frequencies while the vertical axis refers to the modulated frequencies. From the comodulograms, one can understand that the basic modulators of intrinsic activity are mainly δ, θ, α_1_, and α_2_ brain rhythms.

The outcome of this novel approach is demonstrated in Figure 3. The colored lines illustrate the fluctuation of the preferred coupling modes for three pairs of MEG sources from participant 1 (Figure 3a). The color codes the strength of the iPLV/PAC connectivity estimator while the *y*‐axis refers to one of the 36 possible coupling modes. The colored 2D matrices are called comodulograms and summarize the probability distribution of each coupling mode for a single pair of MEG ROIs (Figure 3b). Figure 3a illustrates the core of our methodology that explains how we integrate the dominant coupling modes into a single dynamic functional connectivity graph. The main outcome of this model is a sequence of dominant coupling modes between every pair of neuromagnetic sources across experimental time. Dominant coupling modes can be seen as the basic letters of neural transmission that can form a “word” for neural information exchanged between two sources.

We illustrate the dynamic reconfiguration of dominant coupling modes, across experimental time, for four pairs of sources from a single subject. Each subplot in Figure 3a illustrates the richness of information in neuromagnetic source connectivity time series in terms of the fluctuations of dominant coupling modes over and above mere coupling strength.

In Figure 3, we showed the first 2 s out of 300 s for four pairs of sources. Our DoCM model untangled the dominant coupling mode per pair of brain areas at every snapshot of the dynamic connectivity analysis. These sequences of DoCM were used to construct the comodulograms that tabulate the probability distribution of DoCM across experimental time per every pair of brain areas (see Figure 3, right column).

## 3 MODELLING THE DYNAMIC RECONFIGURATION OF DOMINANT COUPLING MODES VIA MARKOVIAN MODELS (EXPERIMENT 1)

We modeled the time series that describes the temporal evolution of the dominant coupling mode per pair of sources with a discrete Hidden Markovian Model (dHMM; Figures 2 and 3). For every time series called hereafter DoCM^ts^, we can estimate the probability distribution (PD) of DoCM. PD is a vector of size 36 that quantifies the PD of DoCM across experimental time which equals 2991 temporal segments. We estimated PD for every pair of virtual sources.

For every DoCM^ts^, a dHMM was used to model each DoCM^ts^ using the Expectation Maximization algorithm (Baum–Welch method). With this approach, for every DoCM^ts^ and independently for each subject, we searched for the best model described by the transition matrix, the priors, and the observation matrix by minimizing the log‐likelihood between the original system and the one modeled via the dHMM. For that purpose, dHMMs were trained using the Baum–Welch algorithm (for further details, see Section 1 in the Supporting Information).

We optimized the number of states for each DoCM by minimizing the error between the trained dHMM and the original data. This leads to 90 × (90 – 1)/2 = 4005 training sets for each subject and scan sessions. Each subject is a separate class *k* = 40 and our goal is to detect the subset of MEG source pairs that can accurately detect each subject compared to the rest.

The procedure of brain fingerprinting based on the estimation of probability distribution (PD) of dominant coupling modes over the DoCM^ts^ to increase recognition accuracy can be summarized as follows.

The following steps were repeated separately for each DoCM^ts^.

**FIGURE 4:**
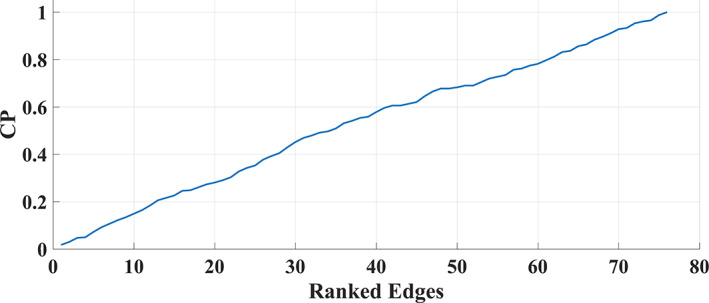
Step‐wise classification performance (CP) of the brain‐fingerprinting for the 76 selected edges.

1. PD is a vector of size 36 that quantifies the probability distribution of DoCM across experimental time which equals 2991 temporal segments.
2. In our case, we estimated PD across six epochs over every time series of size 2991 temporal segments {1–500, 501–1000, 1001–1500, 1501–2000, 2001–2500, 2501–2991} and between every pair of sources. The outcome of this preprocessing step is a feature matrix of size (FM): 6 (epochs) × 36 (no of dominant coupling modes).
3. We trained one dHMM model over every FM related to a pair of sources (*n* = 4005) per subject (*k* = 40) independently per subject from the first scan session.
4. To classify an incoming sequence of DoCM, we computed the log‐likelihood that every *k* model gives to the test sequence derived from the second session. if the *k*'th model is the most likely, then declare the class of the sequence to be class k (subject id).
5. We followed the aforementioned procedure for every 4005 time series and explicitly for every 4005 PD matrices.
6. From this procedure, DoCM^ts^ were ranked according to their discriminative power (performance) to separate participants from the remainder of the sample.
7. This procedure was repeated iteratively with the main scope of aggregating the most important DoCM^ts^ such as to increase the classification accuracy. Then, we added the second DoCM^ts^, and we used the sum of log‐likelihood from the two time series as a way to declare the class *k* of both sequences and so forth. This procedure was followed till reaching a plateau for the identification accuracy.
8. Seventy‐six connections succeeded to discriminate every subject over the rest and the related topology is given in Figure 6a–d. Figure 4 illustrates how the integration of the selected edges improved the classification performance (CP) of brain fingerprinting.
9. Figure 7 illustrates the sum of log‐likelihood outcome using the 76 training dHMM models from every subject, related to each of the 76 selected DoCM^ts^ from the first scan session, with the 76 testing sequences of DoCM^ts^ for every *k* − 1 subject.

## 4 FLEXIBILITY INDEX BASED ON THE DYNAMIC RECONFIGURATION OF DOMINANT INTRINSIC COUPLING MODES

A novel flexibility index (FI) based on the dynamic reconfiguration of DoCM has been applied to the first cohort. FI is based on the concept that variability of connectivity patterns (i.e., more frequent switches between DoCM), captures the brain's flexibility.

We briefly describe its definition in the following section.

The outcome of statistical filtering was two 3D matrices per subject and condition each one with dimensions: 2991 (temporal segments) × 90 (sources) × 90 (sources). The first one keeps the weights of the survived functional connections while the second tabulates with an integer the dominant coupling mode.

From the second 3D matrix, we can estimate the stability of functional connections across time in terms of the DoCM. This estimator is called flexibility index (FI) and encounters for each pair of MEG sources how many times a DoCM changes between two consecutive temporal segments. FI is defined for every pair of virtual sources and in a global manner as follows:

(6) $${\mathrm{FI}}^{\mathrm{MEG}}\text{(Sources, Sources)}=\begin{array}{l} \frac{1}{1-T}\sum_{s=1}^{T-1}\sum_{{\text{source}}_{1}=1}^{\text{Sources}}\sum_{{\text{source}}_{2}={\text{source}}_{1}\;+\;1}^{\text{Sources}}\delta (\text{DoCM}\left(T,{\text{source}}_{1},{\text{source}}_{2}\right),\text{DoCM}\left(T+1,{\text{source}}_{1},{\text{source}}_{2}\right)) \end{array}$$

(7) $${\mathrm{FI}}_{\text{GLOBAL}}^{\mathrm{MEG}}=\frac{{\mathrm{FI}}^{\mathrm{MEG}}}{\left(\text{Sources}\times \left(\text{Sources‐1}\right)\right)/2}$$

where *T* = 2991 and Sources = 90.

We have proposed this FI as a novel temporal variability measure that may be a suitable indicator of the flexibility of a brain region, and could potentially be used to predict the outcome of learning or to demonstrate changes due to disorders. Our recent fMRI‐based dynamic functional connectivity study (Dimitriadis, 2021; Palmigiano et al., 2017; Sorrentino, Rucco, Baselice, et al., 2021) has shown that FI predicts memory.

## 5 OPTIMIZATION OF PARAMETERS FOR THE SLIDING WINDOW APPROACH

The basic parameters of a time‐varying approach based on the sliding window method are the width of the sliding window and the stepping criterion that defines the moving of the window to the next temporal segment. We optimized the basic parameters of the sliding‐window time‐varying approach {width of time‐window, stepping criterion} based on the repeatability of flexibility index (FI) using the dataset from the first cohort. The objective criterion was to increase the correlation of network‐wise FI between the two sessions. This is a significant criterion since in our study we mixed all the different coupling modes into a single dynamic functional brain network. With this procedure, we decided to identify a repeatable frequency of temporal changes of dominant coupling modes across the network, as a way to optimize the sliding window and the stepping criterion but without bias the selection on the connection level. Additionally, the coupling strength of every coupling mode would change by changing the width of the temporal window and also the stepping criterion. However, in our approach, we care about the repeatability of the frequency of temporal changes of dominant coupling modes across the network and not about their coupling strength.

We employed a large set of settings for the width of temporal window {0.5, 0.75, 1, 1.25, 1.5, 1.75, 2, 2.25, 2.5, 2.75, 3} s and stepping criterion {50, 100, 150, 200, 250, 300, 350, 400, 450, 500} ms. This procedure yielded a width of 1 s and a stepping criterion of 100 ms as best settings, based on the optimized repeatability of FI (Figure 6).

**FIGURE 5:**
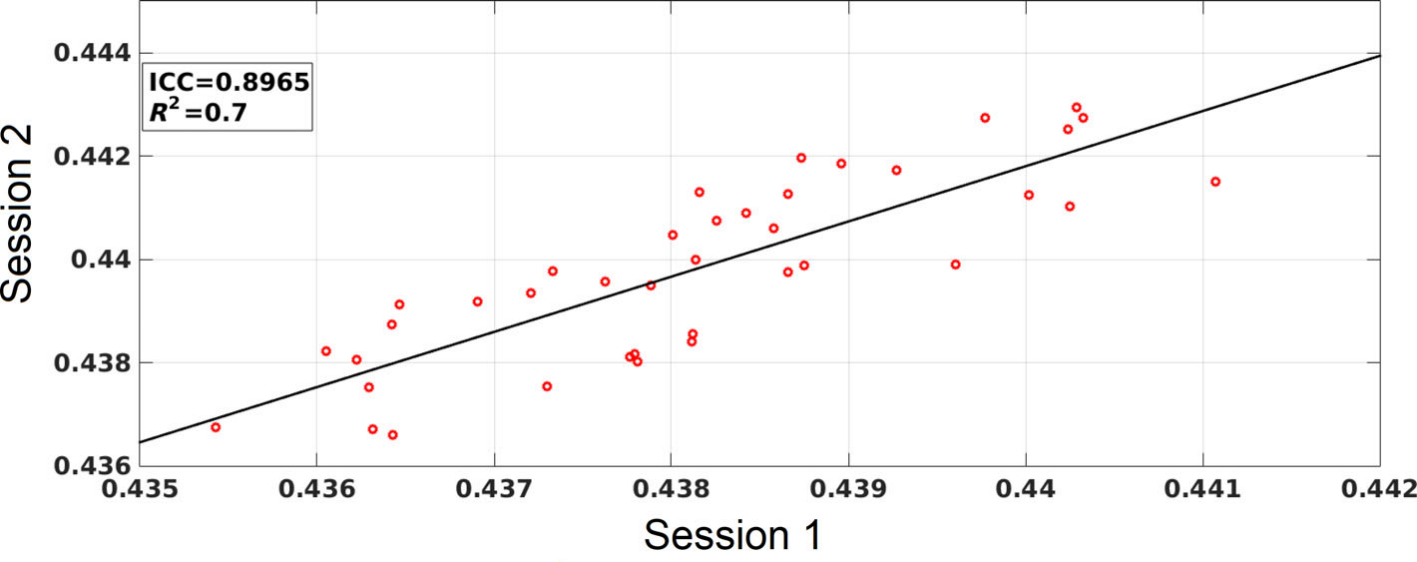
Repeatability of FI across the repeat MEG resting‐state cohort.

Figure 5 illustrates the correlation of FI across sessions 1 and 2 for the 40 subjects. The intra‐class correlation coefficient is 0.89, the *R* ^2^ is 0.7 and the correlation coefficient is 0.8569.

## 6 EXTERNAL VALIDATION OF THE IDENTIFICATION PROCESS IN A SECOND COHORT (EXPERIMENT 2)

To validate our identification scheme, we repeated the whole preprocessing analysis in the second cohort of *N* = 183 subjects. Our goal was to blindly identify the participants that were common to both cohorts (first cohort of 40 repeat scans and the second cohort of *N* = 183 subjects). Author SID did not know how many participants from the test–retest study participated in the population study. The main goal of this external blind identification step was to identify the correct number and identity of subjects from the first cohort that have a third scan in the second cohort.

**FIGURE 6:**
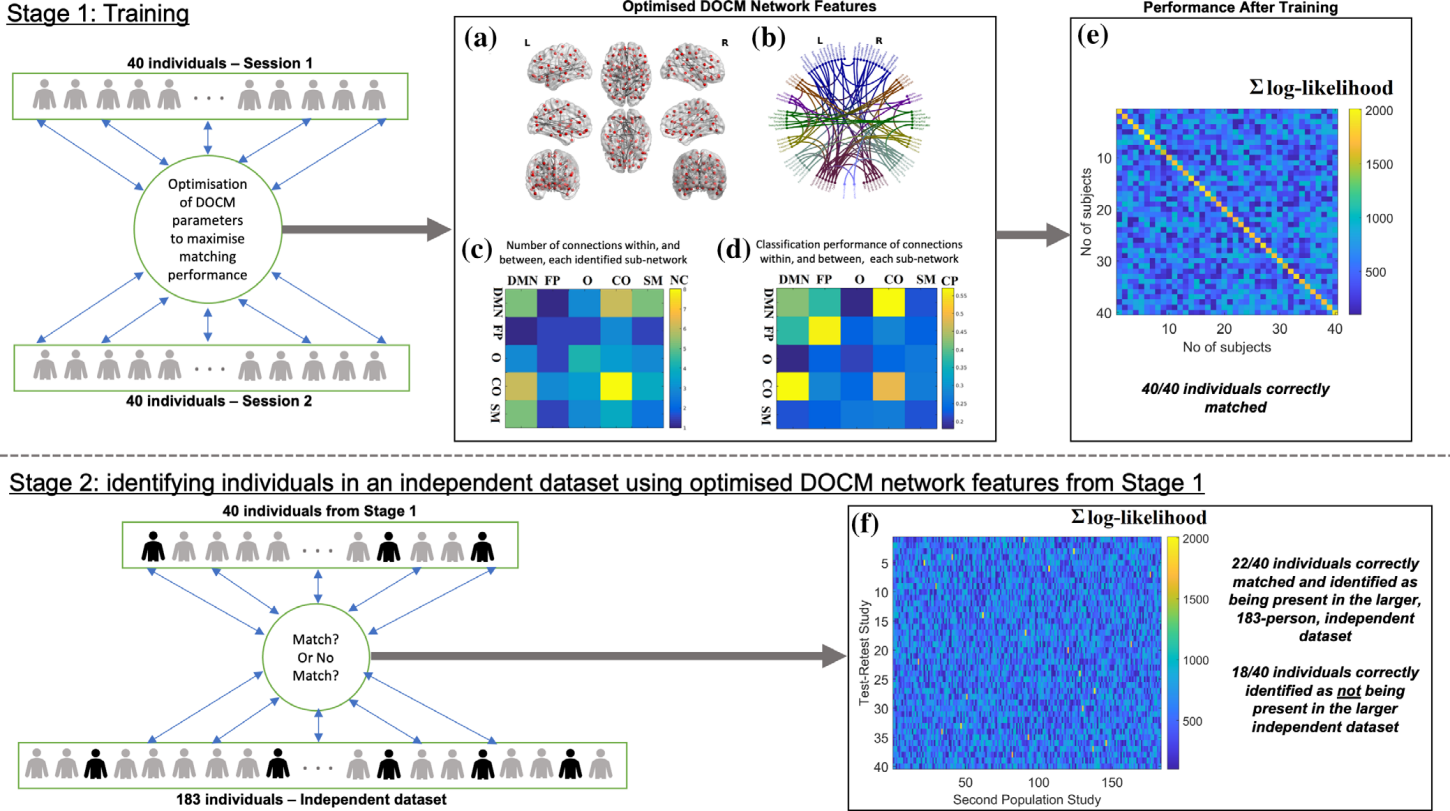
Results of the two‐stage analysis procedure. The upper panel (Stage 1) shows the identification training using 40 participants with repeat resting‐state MEG data. The lower panel (Stage 2) shows the blind matching, or nonmatching, of these 40 people to an independent dataset of 183 people. Panels (a) and (b) show the nodes and connections that were identified as being important DoCM network features for the initial training. In (a), these are plotted on a 3D template brain representation, while in (b), the same connections are shown on a circular representation of the 90 AAL atlas regions. In (c), the distribution of the 76 connections identified as part of the multi‐parametric brain fingerprinting approach to the five sub‐networks is shown. Each color encodes the total number of connections (NC) related to the identified 76 pairs within and between the five sub‐networks. CO, cingulo‐opercular; DMN, default mode network; FP, fronto‐parietal; O, occipital; SM, sensory motor). In (d), the classification performance of each of the same sub‐networks is shown. Each color encodes the classification performance (CP) of the 76 connections integrated within and between the five networks. (e) shows the performance of the matching procedure as a similarity matrix illustrating the summation of log‐likelihood across 76 training discrete Hidden Markov Models (dHMM) models from the first dataset of each subject (*x*‐axis) and from the second dataset of each subject (*y*‐axis). (f) shows the performance of the independent matching test as a similarity matrix, showing the sum of log‐likelihoods across 76 training dHMM models from every subject of the test–retest study and each set of 76 tested sequences of every subject from the population study. Yellow pixels in this matrix represent the successful identification of subjects from the first test–retest study that indeed participated in the second study.

For that reason, we have to extract a decision‐making scheme from the sum of log‐likelihood as derived by using the 76 training dHMM models from every subject, related to each of the 76 selected DoCM^ts^ from the first scan session, with the testing sequences for every *k* − 1 subjects (step 9 described in previous section 3). Figure 6e illustrates the similarity matrix that encapsulates each pair‐wise sum of log‐likelihood. Based on the similarity matrix shown in Figure 6e, we estimated the threshold values important for the external validation task. We estimated the threshold^1^ 574.01 ± 15.51 from the off‐diagonal values and the threshold^2^ 1754.90 ± 116.55 from the diagonal values from Figure 6e. Both thresholds are important to identify the unknown number of subjects from the first test–retest study that participated in the second large cohort.

**FIGURE 7:**
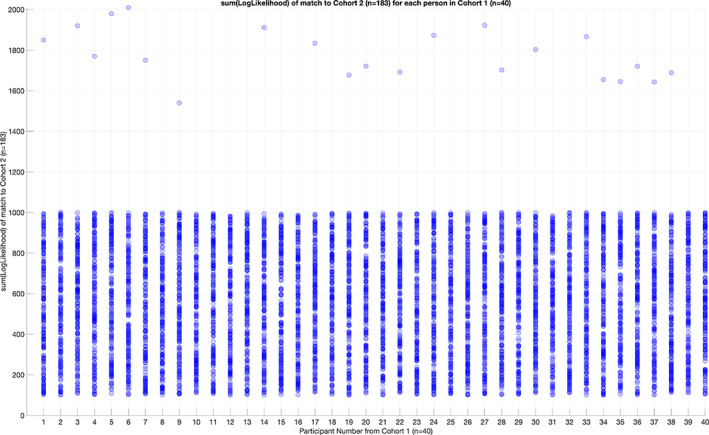
Performance of cross‐experiment identification. For each of the 40 participants in Cohort 1, the sum(LogLikelihood) is plotted for each match to the 183 people in the second cohort. For 18 of the participants, this is a distribution of values between 100 and 1000, representing no match, that is, the algorithm has correctly estimated that these 18 people were not in the second cohort. For 22 of the 40 participants, a single sum(LogLikelihood) is seen that exceeds a value of 1400. This represents a successful match between Cohort 1 and Cohort 2.

**FIGURE 8:**
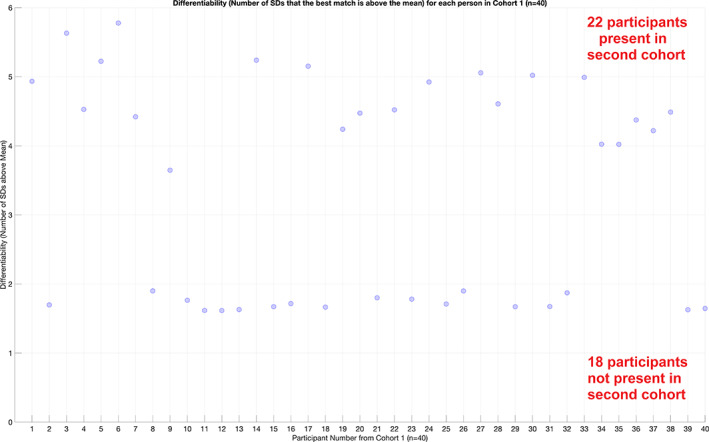
Differentiability scores (da Silva Castanheira et al., 2021), for each of the original 40 participants, when matching is attempted in cohort 2 (*N* = 183). For those 22 participants present in the second cohort, the mean score is 4.7 ± 0.5. For those 18 participants not present in the second cohort, the score is 1.7 ± 0.1.

The 76 trained dHMM models for every DoCM^ts^ derived from the first scan session and applied independently per subject were used for testing every set of 76 DoCM^ts^ from the second population cohort of 183 subjects. Applying the threshold as derived from the similarity matrix (Figure 6e) and it was stated above, we decided if a subject from the first study participated also in the second cohort. Figure 6f illustrates the similarity matrix that tabulates the sum of log‐likelihood between every pair of subjects between the two studies. The performance of cross‐experiment identification with the relevant sum of log‐likelihood estimated between the two cohorts is shown in Figure 7. Figure 8 illustrates the Differentiability scores, for each of the original 40 participants, when matching is attempted in cohort 2 (*N* = 183).

## 7 VALIDATION OF THE DOCM ACROSS ALTERNATIVE SCENARIOS

In order to strengthen the outcome of our research approach based on DoCM, we applied the following experiments answering the following questions. The following experiments were applied to both cohorts as with the original DoCM approach.

1. Does the set of 76 connections showed in Figure 6b is unique across surrogate sets of 76 connections across the total set of 4005?

To answer to this type of question, we randomly selected 1000 random sets of 76 connections sampling the complete set of 4005 possible pairs, and presenting the outcome of this approach following the same procedure.

2 Does the power spectrum could show similar performance with the semantic information of DoCM?

We employed Welch's method to derive power spectrum density (PSD) estimates for each ROI, using time windows of 2 s with 50% overlap sled over all ROI representative time‐series (Welch, 1967). The resulting frequency range of PSDs was 0–90 Hz, with a frequency resolution of 0.5 Hz. The following procedure under the brain fingerprinting framework has been realized independently for each of the eight studying frequency bands. We created a PSD profile from every subject and scan session by concatenating the ROI‐based PSD profile into a single vector of features.

We used the absolute value of Pearson's correlation coefficient to quantify the correlation between every PSD profile of every subject from scan session 1 with the same PSD profile of all the subjects from scan session 2. This procedure should be run exhaustively between every subjects' PSD profile from scan session 1 with every subjects' PSD profile from scan session 2 producing a correlation vector of size [1 × 40 (subjects)]. The index of the column featuring the largest absolute correlation coefficient determined the predicted (anonymous) identity of the individual in the second session cohort. Thus, if a given individual's PSD profile from the first dataset were most correlated to the PSD profile from their second dataset, the individual would be correctly differentiated. This procedure will produce a binary vector of size [1 × 40] with 1 s where an individual was correctly differentiated from the rest of the cohort, and 0 s where is not correctly classified. By summing the columns of this vector and dividing by the total amount of subjects (here 40), we can access the classification performance of PSD from the test‐retest cohort. Similarly, the classification performance was estimated for the identification of the 22 subjects in the second validation cohort.

3 Does the static connectivity network could show similar performance with the semantic information of DoCM?

We constructed static frequency‐dependent functional brain networks of size [90 (ROIs) × 90 (ROIs)] per subject and scan session. Similarly, as in the PSD analysis, we vectorised the upper triangular of the brain networks producing a vector of features of size 4005 which are the possible pair‐wise connections between every possible pair of the 90 ROIs (90 × 89/2 = 4005 pairs). We followed the same brain fingerprinting framework as described above. This procedure has been realized independently for each of the eight studying frequency bands.

4 Does the strength of dynamic connectivity network could show similar performance with the semantic information of DoCM?

In our approach, we kept and analyzed with dHMM method, the semantic information tabulated in 3D matrices of size The outcome of DoCM was two 3D matrices per subject and condition each one with dimensions: 2991 (temporal segments) × 90 (sources) × 90 (sources). The first one keeps the weights of the survived functional connections while the second tabulates with an integer the dominant coupling mode. Both 3D matrices constitute the integrated dynamic functional connectivity graphs (iDFCG). Here, we will use the first 3D matrix that tabulates the functional coupling strength of the dominant coupling modes as a way to test its brain fingerprinting validity versus the proposed approach. The second and third dimension of the 3D matrix that refers to a snapshot of the dynamic functional connectivity graph was vectorised as in the static approach transforming the 3D matrix into a 2D matrix of size [2991 × 4005]. We followed the same brain fingerprinting framework as described above but we adopted the Euclidean distance as a proper distance metric to measure the similarity of two 2D matrices.

## 8 RESULTS

### 8.1 Stage 1: Identification of a neuromagnetic fingerprint from the dynamic reconfiguration of dominant coupling modes

In the first stage, we analyzed the test–retest dataset, performing a 2D grid search using the whole‐brain connectivity matrix, with no a priori restriction over specific subnetworks. Τhe differentiability score for the test–retest dataset was 4.3 ± 0.4 while the differentiation accuracy was 100% (Figure 6e) for the 40 subjects. Figure 6e illustrates the sum of log‐likelihood outcome using the 76 training dHMM models from every subject related to each of the 76 selected DoCM^ts^ from the first MEG session with the 76 sequences of DoCM^ts^ from the second MEG session for each subject. The in‐diagonal of this matrix showed a high concordance between the two MEG sessions for every subject and a low likelihood between every pair of subjects (off‐diagonal) supporting the absolute accuracy of the classification. Based on the similarity matrix shown in Figure 6e, we estimated the threshold values important for the external validation task. We estimated the threshold^1^ 574.01 ± 15.51 from the off‐diagonal values and the threshold^2^ 1754.90 ± 116.55 from the diagonal values from Figure 6e. Both thresholds constituted the range of *sum*(*LogLikelihood*) *values* that are important to identify the subjects from the first test–retest study that participated in the second large cohort.

#### 8.1.1 Subnetwork identification based on the dynamic reconfiguration of dominant coupling modes

We ranked all the pairs of anatomical modes according to their discriminative power and then integrated step‐wise the pairs if they improved the discrimination accuracy further. The topology of these connections is given in Figure 6a,b. The distribution of these pairs within and between the five brain networks can be seen in Figure 6c,d. Major contributions were located within the cingulo‐opercular network (CO: 8 connections) and between the default‐mode network (DMN) and the CO (DMN‐CO: 7 connections) and the sensorimotor (SM) network (DMN‐SM: 6 connections). Βased on the probability distribution of the selected 76 time‐series between pairs of ROIs, we revealed that the major frequency contributors both within and cross‐frequency coupling modes were in descending order α_1_, δ, α_2_, and θ.

After training optimization, we achieved 100% identification of the independent second MEG measurement of the 40 participants of the repeat cohort using 76 pairs of anatomical nodes from (90 × 89/2 = 4005) possible connections.

#### 8.1.2 Quantifying edgewise contribution to brain identification

To quantify the extent to which the 76 pairs contributed to the fingerprinting of the 40 subjects, we repeated the same classification procedure by integrating the connections either within or between the five brain networks. The dynamic reconfiguration of DoCM for every pair of connections that group together in the same brain network was used as a unique pool of features. We did not average the evolution of DoCM for pairs grouped within the same network or between the networks. A pair can connect two brain areas that are both located on the same brain network (5 total cases) or each brain area is located in different brain networks. This gives 5 × (5–1)/2 = 10 pair‐wise combinations of the brain networks and one configuration for each brain network giving us a total number of 15 runs. We repeated the same classification identification methodology independently for the 15 total cases. We finally, ranked the performance of the 15 cases to reveal the highest contribution from each one. Figure 6d illustrates the classification performance of the identification for each of the 15 cases. The best performance for individual discrimination was achieved at 57.5% (23 out of 40 subjects;) for the pair of DMN‐CO and by the within‐FP DoCM with 57.5% (23 out of 40 subjects), the DoCM within the CO subnetwork with 50% (20 out of 40 subjects), the DoCM within the DMN subnetwork with 42.5% (17 out of 40 subjects) and the DoCM of the DMN‐FP integration with 37.5% (15 out of 40 subjects), O with 22.5% (9 out of 40 subjects) and SM with 25% (10 out of 40 subjects) contribute the least to individual subject identification.

### 8.2 Stage 2: Testing the neuromagnetic brain fingerprinting approach in a second population study

To further validate our approach of brain fingerprinting in a second dataset (external validation), we applied the same framework, using the same subnetwork of 76 connections, to MEG resting state datasets from 183 subjects who had participated in a multi‐modal study in CUBRIC as part of the creation of a large normative neuroimaging database (Buzsáki & Wang, 2012). With this approach, we replicate the same methodology and also the relevance of the nodes highlighted in the previous stage. We succeeded to recover both the number (*N* = 22) and the identity of all subjects from our cohort of 40 (Experiment 1) who were involved also in this second study (Figure 6f). Yellow pixels in this 2D similarity matrix correctly identify the 22 subjects who also took part in the second study. Figure 7 illustrates the performance of this cross‐experiment identification. In Figure 7, we showed the *sum*(*LogLikelihood*) of each of the 40 subjects from the first cohort matched to the 183 subjects from the second cohort. For 22 out of the 40 participants, a single sum(LogLikelihood) is seen that exceeds a value of 1400 that supports the absolute differentiation accuracy of 100%. For unsuccessful matches, a uniform distribution between 100 and 1000 can be seen, while in contrast, successful matches show a clear separation in having values above 1400. Figure 8 illustrates the Differentiability scores for both the identified and not identified subjects of the test–retest dataset. For those 22 participants present in the second cohort, the mean score is 4.7 ± 0.5 (Figure 8). For those 18 participants unpresented in the second cohort, the score is 1.7 ± 0.1 (Figure 8).

#### 8.2.1 Brain fingerprinting with surrogates of 76 tuples of DoCM profiles

The average performance of randomly selected 1000 random sets of 76 connections across the 4005 possible pairs was 9.43% ± 3.62 for the repeat scan cohort (dataset 1), and 4.54% ± 3.12 for the validation cohort (dataset 2).

#### 8.2.2 Brain fingerprinting with PSD, static, and dynamic functional connectivity network profiles

The performance of PSD approach in both repeat and validation cohorts is showed in the first row of Table 2. The highest performance was achieved in the repeat cohort in the γ sub‐bands. Similarly, the performance of the static functional connectivity approach in both cohorts is tabulated in the second row of Table 2. Interestingly, the highest performance was achieved for the repeat cohort in γ sub‐bands. Finally, the performance of the integrated dynamic functional connectivity graphs (iDFCG) was 75.00 for the repeat cohort and 77.27 for the validation cohort.

**TABLE 2:** Performance of power spectrum, static and dynamic functional connectivity network in both repeat and validation cohorts.

	*δ*	*θ*	*α*_1_	*α*_2_	*β*_1_	*β*_2_	*γ*_1_	*γ*_2_
*Spectral*								
Repeat cohort	42.50	55.00	52.50	55.00	57.50	52.50	67.50	65.00
Validation cohort	27.27	31.82	36.36	36.36	40.91	45.45	40.91	45.45
*Static connectome*								
Repeat cohort	47.50	57.50	55.00	57.50	62.50	57.50	70.00	72.50
Validation cohort	31.82	36.36	45.45	50.00	63.64	59.09	68.18	72.73

## 9 DISCUSSION

In the present study, we show that an individual's neural dynamics measured with MEG, formalized as a profile of the reconfiguration of dominant coupling modes between brain nodes, constitutes a reliable and unique neurophysiological “fingerprint.” We demonstrate that this novel signal processing approach allows the identification of an individual from a group of subjects only based on the fluctuations of dominant coupling modes of a subnetwork of 76 connections. We validated the identification accuracy strategy based on the DoCM in a second dataset of *N* = 183 subjects by accurately identifying the subjects of the first scan cohort who participated in the second experiment.

To further validate the importance of the DoCM model, we followed specific experiments. We first showed that the set of 76 pairs selected via our approach is a unique set and no other set of randomly selected 76 pairs out of 4005 can produce similar results. The PSD and the frequency‐dependent static functional connectivity network profiles both showed a low performance in both cohorts across the frequency bands. Importantly, the iDFCG profile which tabulates the weights of the DoCM showed the highest performance reaching 75.00 for the repeat cohort and 77.27 for the validation cohort. These findings showed that only the semantic information of DoCM that was modeled via the dHMM succeeded in the absolute performance (100) in both cohorts and especially in the validation cohort.

The intra‐individual consistency of functional brain networks has been highlighted in the resting‐state with both static (Colclough et al., 2016) and dynamic approaches (Abrol et al., 2017; Dimitriadis, López, et al., 2018). However, our study was the first that explored the multiplexity of human brain dynamics by incorporating both within‐ and between‐frequency coupling mechanisms into a single dynamic functional connectivity graph (iDFCG). Whereas the majority of previous studies of intra‐individual consistency analyzed functional connectivity only between predefined brain areas, we took a whole‐brain approach which captures the information flow between brain areas more comprehensively. The reliability of individual functional connectivity patterns of human brain dynamics is relevant to individual differences in cognition, personality, and behavior (Hearne et al., 2016; Kanai & Rees, 2011). Moreover, our results suggest that an individual's dynamic dominant coupling mode profile might be used as a unique subject‐specific descriptor of brain health. Our results underline the potential of MEG and oscillation‐based dynamic connectivity to build novel oscillatory neuromarkers (van Pelt et al., 2012) that can eventually be used to personalize the diagnosis and treatment of mental and neurological disorders and hence improve the outcome of an intervention in clinical practice. For example, a recent study employed a large MEG dataset to explore if brief segments of frequency‐dependent brain activity enable individual differentiation (da Silva Castanheira et al., 2021). They reported a high identification score of 98%. Another study adopted an open MEG dataset from the Human Connectome project where participants underwent three recording sessions within a single day (Sareen et al., 2021). The authors applied a static connectivity analysis adopting both phase and amplitude‐based measures with and without spatial correction methods and in various frequency bands. They showed that all these factors influenced the MEG fingerprinting performance. The identification score of 98% was detected in phase‐coupling methods, in central frequency bands (alpha and beta), and in the visual, frontoparietal, dorsal‐attention, and default‐mode networks (Sareen et al., 2021).

### 9.1 Anatomic loci of distinguishing dynamic dominant coupling modes features

Our data‐driven approach, based on the treatment of the dynamic dominant coupling profile of each pair of brain areas as a Markovian Chain, revealed a small set of connections that accurately identify each individual over the whole set, with the main contributions coming from connectivity between the DMN and the CO and the FP, and within these three networks. The DMN‐CO‐FPN network thus creates a strong backbone for the unique characterization of the spatiotemporal profile of DoCM of each individual.

The importance of the frontoparietal network for individual functional connectivity profiles is consistent with the presumed individual specificity of high‐order association networks that are most recent in evolutionary terms and demonstrate the highest inter‐subject variability (Cole et al., 2012; Kanai & Rees, 2011; van Pelt et al., 2012). Nodes located in the frontoparietal network (FPN) have been identified as flexible hubs that adjust to the requirements and demands of multi‐task activity (Cole et al., 2014). Moreover, the complementary connections that start from the FPN to other areas of the brain, such as the DMN, are consistent with the role of large‐scale coordination of human brain activity (Martuzzi et al., 2010; Smith et al., 2009).

Previous studies in both structural and functional neuroimaging have linked the properties of the FPN to the construction of fluid intelligence (Cole et al., 2014; Smith et al., 2009). Moreover, abnormal functional connectivity in the FPN has been linked to many neuropsychiatric diseases (Preusse et al., 2011; Sheffield et al., 2015; Tschentscher et al., 2017). FPN and CON are hypothesized to support top‐down control of executive functioning and for that reason can be seen as potential drivers of cognitive impairment in diseases such as schizophrenia (Cetin et al., 2016; Gross, 2019; Uhlhaas & Singer, 2012). The DMN–FPN–CON are thought to interact and together control attention, working memory, decision‐making, and other higher‐level cognitive operations (Cetin et al., 2016; Tschentscher et al., 2017).

Adding the time dimension into the analysis of brain connectomes yields “chronnectomes” based on network metrics that allow a dynamic view of functional coupling modes. In the present study, we demonstrated that fluctuations of dominant coupling modes between brain networks are *oscillatory fingerprints* of individualized chronnectomes in healthy individuals (Finn & Todd Constable, 2016; Horn et al., 2017). Brain oscillations are amongst the neural phenotypes with the highest heritability (Begleiter & Porjesz, 2006; van Beijsterveldt & Boomsma, 1994). For this reason, brain rhythms have long been explored as potential endophenotypes of cognition and complex genetic disorders such as autism (David et al., 2016), schizophrenia and bipolar disorder (Başar et al., 2016), or Alzheimer's disease (Pusil et al., 2019) but progress has been hampered by a lack of reliabl, individually specific neuroelectric or neuromagnetic metrics. Our discovery of neuromagnetic fingerprints based on dominant coupling modes can thus become a signature of individual brain health and a marker for the progression of the disease and also a validated substrate for the design of novel personalized treatments (Finn & Todd Constable, 2016; Uhlhaas et al., 2017).

MEG resting‐state functional connectivity patterns are stable across life time within the subjects. A study took the advantage of this observation to explore the similarity of whole brain functional connectivity patterns to identify monozygotic twin pairs. They succeeded an identification rate of 75% showing large similarities in brain connectivity patterns between two genetically identical individuals even after 60 years of life or more (Demuru et al., 2017). Another study proposed the identifiability score and the general brain fingerprinting framework as a way to define clinical connectome fingerprints relevant to cognitive decline (Sorrentino, Rucco, Lardone, et al., 2021). In another study, the authors proposed the clinical connectome fingerprint (CCF) approach where they showed a reduction of the identifiability score in the cohort with Parkinson's disease in beta band which was also proportional to the motor impairment (Lopez et al., 2023). The clinical utility of CCF was demonstrated by its ability to predict the individual motor impairment in patients affected by ALS (Romano et al., 2022). Another study explored the uniqueness of dynamic functional connectivity patterns across different temporal scales (van de Ville et al., 2021).

Brain fingerprinting in the resting‐state and the identification of personalized brain subnetworks can thus be highly relevant for understanding the biological basis of personality and cognitive traits. A recent study based on fMRI resting‐state recordings and adopting a chronnectomic approach not only reported a high accuracy in identifying subjects, but also that the discriminative features predicted cognitive performance in domains such as fluid intelligence and executive function (Liu et al., 2018). Another study reported that task‐evoked brain activity estimated over brain regions defined by the resting‐state networks explains the link between resting‐state functional connectivity and cognitive task activations (Jiang et al., 2020).

## 10 METHODOLOGICAL CONSIDERATIONS

It is important to mention here that our attempt was not to exhaustively explored how alternative graph construction scenarios can alter the final outcome of our research. We decided to investigate the multiplexity of resting‐state brain oscillations in the phase domain. Similar analysis could be followed in the amplitude domain by adopting, for example, the correlation of the envelope (Colclough et al., 2016). In our study, we adopted a famous atlas; the AAL which is highly used in MEG studies, and for that reason, many researchers can compare the findings with their own findings. We also adapted our approach to define the representative time series by weighting differently every voxel time series per ROI. However, there are alternative approaches on how one can define the representative time series. The first class is by collecting one voxel time series that (a) is located in the centroid of the convex hull that orients the brain area (Centroid method) and (b) also the one that encompasses the maximum power spectrum (Power Spectrum method). The second class is by summarizing the activity from all the voxel time series (a) by applying PCA on the voxel time‐series and extracting the first principal component but with the drawback that the percentage of variance explained by the first principal component compared to the whole set of voxel time series could be different across the population or groups for specific ROIs, (b) by getting the mean across the voxel time series which is a common technique mainly in fMRI (Mean method), (c) by weighting every voxel time series according to its complementarity (Interpolation method–our approach). In our approach, the contribution of every voxel time series to the representative time series changes across experimental time and temporal segments. The definition of the representative time series per ROI could affect the brain connectivity in both static (Dimitriadis, Routley, et al., 2018) and dynamic scenarios and also in the multiplex approach, but this is out of the scope of our study.

## 11 CONCLUSION

People can be identified through characteristic patterns of dynamic changes in functional connectivity during rest. This oscillatory fingerprint, derived from neuromagnetic data, was mainly driven by functional connectivity in and between frontoparietal, default mode, and cinguloopercular networks, highlighting the role of these networks in individual attributes such as intelligence and personality. Such individually specific patterns of neural dynamics can help unravel the neural mechanisms of stable individual traits such as personality features and intelligence and may in the future provide the basis for personalized diagnostics of changes in brain health.

## CONFLICT OF INTEREST STATEMENT

The authors declare that they do not have any conflict of interest.

## Supporting information

Supporting Information

## Data Availability

The data that support the findings of this study are available from Professor Krish D. Singh (singhkd@cardiff.ac.uk) upon reasonable request.
