# Supplementary material for: Multiplexity of human brain oscillations as a personal brain signature
Source: Hum Brain Mapp. 2023 Sep 5;44(17):5624–40. doi: 10.1002/hbm.26466 (PMC10619372; doi:10.1002/hbm.26466)
Supplement: Supplementary file 1 — Supporting Information [file HBM-44-5624-s001.pdf]

Multiplexity of Human Brain Oscillations as a Personal Brain Signature

Abbreviated Title: A Personalized Multiplex Human Brain Signature

S. I. Dimitriadis[1-3*], B. Routley[1], D.E.J. Linden[1,2,,4], K.D. Singh[1]

[1]Cardiff University Brain Research Imaging Centre, School of Psychology, Cardiff

University, Cardiff, United Kingdom

[2]MRC Centre for Neuropsychiatric Genetics and Genomics, Division of Psychological Medicine and Clinical Neurosciences, School of Medicine, Cardiff University, Cardiff, United Kingdom

[3] Department of Clinical Psychology and Psychobiology, University of Barcelona, Barcelona, Spain

[4]School for Mental Health and Neuroscience, Faculty of Health Medicine and Life Sciences, Maastricht University, Maastricht, the Netherlands

***Corresponding Author:**

Address: Department of Clinical Psychology and psychobiology, University of Barcelona, Passeig Vall d'Hebron 171, 08035 Barcelona, Spain.

Email : DimitriadisS@cardiff.ac.uk ; stidimitriadis@gmail.com

1. Hidden Markov Modeling

Baum-Welch algorithm finds values for HMM parameters that best fit the observed data. For training we need:

- sequence of observations $O_1, O_2, \ldots, O_n$
- sequence of hidden states for these observations $S_1, S_2, \ldots, S_n$

Algorithm tries to estimate transition matrix A and emission matrix B using values of O and S. Since, we don't known the O and S, we assigned a random initial guess of their values. Then, the algorithm iterates using the training DoCM^{ts} and re-calculate both O and S matrices till the convergence of the algorithm.

Expectation maximization

Baum-Welch algorithm is using expectation maximization (EM) approach to find values for A and B.

1. Initialize A and B with some initial values (done only once)
2. Then, the algorithm estimates latent variables $\xi_{ij}(t)$ and $\gamma_i(t)$ using A, B, O and S. This step estimates how much each transition and emission has been employed. This is called the 'estimation step'.
3. Then, the algorithm maximizes A and B matrices employing estimations (latent variables) from previous steps. This is called the 'maximization step'
4. The procedure continues until its convergence

Initial equations

For the estimation of A and B matrices, we used the following formulas (source):

1. A = a_{ij} (probability of transition from hidden state i to hidden state j)= expected number of transitions from hidden state i to state j /expected number of transition from hidden state i
2. B = b_{jk} (probability of observing observation O_k in hidden state j)= expected number of times model is in hidden state j and we observe O_k/ expected number of times in hidden state j

The probability a_{ij} could be defined as the probability of being in hidden state i at time t and in hidden state j at time t+1, given the observation sequence O and the model (source). The described procedure can be graphically showed as follows:

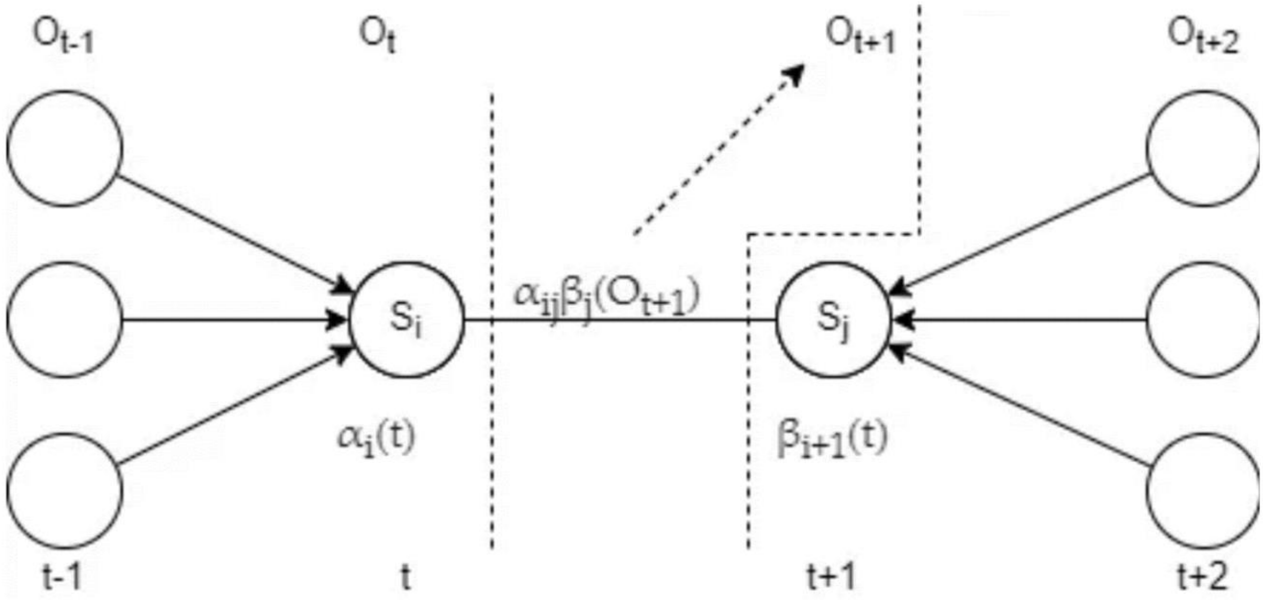

S1. Current step probability with forward, backward and emission probability

In the graph we are at time t, we know probability that we are at the current hidden state S_i (this is forward probability $\alpha_i(t)$), we know hidden probabilities going from hidden state S_j to the end of the sequence using backward probabilities $\beta_{i+1}(t)$. We want to get probability of going from S_i to S_j and given that we have observed O_{t+1} in S_j at t+1.

Here we'll make use of latent variables $\xi_{ij}(t)$ and $\gamma_i(t)$:

- **ξ_{ij}(t)**-probability of transition from hidden state i to hidden state j at time t given observations:

$$\varepsilon_{ij}(t) =)P(X_t = i, X_{t+1} = j|Y, \Theta) = \frac{P(X_t = i, X_{t+1} = j, Y|\Theta)}{P(Y|\Theta)} = \frac{\alpha_i(t) a_{ij} b_j(O_{t+1}) \beta_j(t+1)}{\sum_{i=1}^{M} \sum_{j=1}^{M} \alpha_i(t) a_{ij} b_j(O_{t+1}) \beta_j(t+1)}$$

(1)

Note that denominator P(O|Θ) means probability of the observation sequence O by any path given the model Θ. $\xi_{ij}(t)$ is defined for time t only. We have to sum over all time-steps to get the total joint probability for all the transitions from hidden state i to hidden state j (calculate a_{ij}). This will be our numerator of the equation of a_{ij}. For denominator we could use marginal probability which means the probability of being in state i at time t, whole equation has the following form:

$$a_{ij} = \frac{\sum_{t=1}^{T-1} \varepsilon_{ij}(t)}{\sum_{t=1}^{T-1} \sum_{j=1}^{M} \varepsilon_{ij}(t)} \quad (2)$$

Denominator could be expressed differently and leads to a new latent variable $\gamma_i(t)$:

$$\sum_{j=1}^{M} \varepsilon_{ij}(t) = \frac{\alpha_i(t)\beta_i(t)}{\sum_{i=1}^{M} \alpha_i(t)\beta_i(t)} = \gamma_i(t) \quad (3)$$

$\gamma_i(t)$-probability at given state i at time t given observations. We can use it to calculate a_{ij} (our previous formula for a_{ij} is also valid):

$$a_{ij} = \frac{\sum_{t=1}^{T-1} \varepsilon_{ij}(t)}{\sum_{t=1}^{T-1} \gamma_i(t)} \quad (4)$$

We can use $\gamma_i(t)$ to calculate b_{jk} (which is the probability of a observation O_k from the observations O given hidden state j):

$$b_{jk} = \frac{\sum_{t=1}^{T-1} 1_{O_t=k}\gamma_i(t)}{\sum_{t=1}^{T-1} \gamma_i(t)} \quad (5)$$

Note that $1_{ot=k}$ is an indicator function which has value 1, if observation O_t belongs to class k and 0 if it doesn't.

Expectations and maximization in HMM

Based on the aforementioned equations, we can calculate components separately under the form of EM approach. We have to follow these two steps:

- Calculate expected value of latent variables $\xi_{ij}(t)$ and $\gamma_i(t)$. One can either l initialize A and B randomly or use some previous knowledge if we have it. Here, we used the $DoCM^{ts}$ for every subject's ROI from the scan session 1 to initialize A and B.

- Maximize values of A and B by using equations for a_{ij} and b_{jk}. And go for a next round by using new A and B values for estimating $\xi_{ij}(t)$ and $\gamma_i(t)$.

We have only observations and we start with random guess (or if we have some more information we could use it). We estimate our latent variables which we'll be then used to maximize A and B. At each step we get a better estimation for A and B until improvements are small and algorithm will converge.

2. Nodes to networks mapping

STable 1 tabulates the id, the full name of each brain area's, its name according to AAL (Rolls et al., 2015), the located lobe, the abbreviated name and also the corresponding network used in our study. Here, we used the first 90 areas excluding the cerebellum brain areas (91-116).

The **Default Mode Network (DMN)** involves the following brain areas :

3 4 5 6 7 8 9 10 11 12 13 14 15 16 23 24 25
26 27 28 31 32 33 34 35 36 37 38 39 40 55 56 61 62
65 66 67 68 85 86 87 88 89 90

The Occipital (O) network involves the following brain areas:

43 44 45 46 47 48 49 50 51 52 53 54 55 56

The **Cingulo-Opercular (CO)** network involves the following brain areas:

29,30 31,32, 77,78

The **Sensory-Motor (SM)** network involves the following brain areas:

1 2 19 20 57 58

The **Fronto-Parietal (FP)** network involves the following brain areas:

13 14 15 16 17 18 35 36 59 60 61 62 63 64 65 66 67 68 69 70

STable 1. The anatomical regions defined in each hemisphere and their label in the automated anatomical labeling atlas - AAL (Rolls et al., 2015). Odd/even numbers refer to the left/right homologue brain areas.

ID	Region Description	AAL	Lobe	Abbreviation
1,2	Precentral gyrus	Precentral	Sensorimotor	PreCG
3,4	Superior frontal gyrus,dorsolateral	Frontal_Sup	Frontal	SFG
5,6	Superior frontal gyrus,orbital	Frontal_Sup_Orb	Frontal	SForb
7,8	Middle frontal gyrus	Front_Mid	Frontal	FMid
9,10	Middle frontal gyrus, orbital	Front_Mid_Orb	Frontal	FMorb
11,12	Inferior frontal gyrus, opercular	Front_Inf_Oper	Frontal	IFoper
13,14	Inferior frontal gyrus, triangular	Front_Inf_Tri	Frontal	IFtri
15,16	Inferior frontal gyrus, orbital	Front_Inf_Orb	Frontal	IForb
17,18	Rolandic operculum	Rol_Oper	Frontal	Roloper
19,20	Supplementary motor area	Supp_motor_Area	Sensorimotor	SMA
21,22	Olfactory cortex	Olfactory	Frontal	OLF
23,24	Superior frontal gyrus, medial	Frontal_Sup_Med	Frontal	SFGmedial
25,26	Superior frontal gyrus, medial orbital	Frontal_Med_Orb	Frontal	PFCventmed
27,28	Gyrus rectus	Rectus	Frontal	REC
29,30	Insula	Insula	Subcortical	INS
31,32	Cingulate gyrus, anterior part	Cingulate_Ant	Frontal	ACC
33,34	Cingulate gyrus, mid part	Cingulate_Mid	Frontal	MCC
35,36	Cingulate gyurs, posterior part	Cingulate_Post	Parietal	PCC
37,38	Hippocampus	Hippocampus	Temporal	HIP
39,40	Parahippocampal Gyrus	Parahippocampal	Temporal	PHG
41,42	Amygdala	Amygdala	Subcortical	AMYG
43,44	Calcarine fissure and surrounding cortex (V1)	Calcarine	Occipital	V1
45,46	Cuneus	Cuneus	Occipital	CUN
47,48	Lingual	Lingual	Occipital	LING
49,50	Superior Occipital Gyrus	Occipital_Sup	Occipital	SOG
51,52	Middle Occipital Gyrus	Occipital_Mid	Occipital	MOG
53,54	Inferior Occipital Gyrus	Occipital_Inf	Occipital	IOG
55,56	Fusiform Gyrus	Fusiform	Occipital	FFG
57,58	Postcentral Gyrus	Postcentral	Sensorimotor	PoCG
59,60	Superior parietal Gyrus	Parietal_Sup	Parietal	SPG
61,62	Inferior parietal Gyrus	Parietal_Inf	Parietal	IPG
63,64	Supramarginal Gyrus	Supramarginal	Parietal	SMG
65,66	Angular Gyrus	Angular	Parietal	ANG
67,68	Precuneus	Precuneus	Parietal	PCUN
69,70	Paracentral Lobule	Paracentral_Lobule	Parietal	PCL
71,72	Caudate nucleus	Caudate	Subcortical	CAU
73,74	Lenticular nucleus, Putamen	Putamen	Subcortical	PUT
75,76	Lenticular nucleus, Pallidum	Pallidum	Subcortical	PAL
77,78	Thalamus	Thalamus	Subcortical	THA
79,80	Heschl's Gyrus	Heschl	Temporal	HES
81,82	Superior Temporal Gyrus	Temporal_Sup	Temporal	STG
83,84	Temporal Pole Superior Temporal Gyrus	Temporal_Pole_Sup	Temporal	TPOsup

85,86	Middle Temporal Gyrus	Temporal_Mid	Temporal	MTG
87,88	Temporal Pole<br>Middle Temporal Gyrus	Temporal_Pole_Mid	Temporal	TPOmid
89,90	Inferior Temporal Gyrus	Temporal_Inf	Temporal	ITG

References

1. Rolls, E. T., Joliot, M., and Tzourio-Mazoyer, N. (2015). Implementation of a new parcellation of the orbitofrontal cortex in the automated anatomical labeling atlas. Neuroimage 122, 1–5. doi: 10.1016/j.neuroimage.2015.07.075
